# Human health benefit and burden of the schizophrenia health care pathway in Belgium: paliperidone palmitate long-acting injections

**DOI:** 10.1186/s12913-019-4247-2

**Published:** 2019-06-19

**Authors:** Sam Debaveye, Delphine De Smedt, Bert Heirman, Shane Kavanagh, Jo Dewulf

**Affiliations:** 1 Research Group Environmental Organic Chemistry and Technology (EnVOC), Faculty of Bioscience Engineering, Ghent University, Campus Coupure, Coupure Links 653, B-9000 Ghent, Belgium; 2 Department of Public Health, Ghent University, Campus UZ, De Pintelaan 185, B-9000 Ghent, Belgium; 3 Johnson & Johnson EHS&S, Janssen Pharmaceutica NV, Turnhoutseweg 30, B-2340 Beerse, Belgium; 4 Health Economics, Janssen Pharmaceutica NV, Turnhoutseweg 30, B-2340 Beerse, Belgium

**Keywords:** Life cycle assessment, Environmental sustainability, Pharmaceutical science, Paliperidone palmitate.

## Abstract

**Background:**

Environmental impact assessments of pharmaceuticals typically consider only a part of the pharmaceutical supply chain, e.g. tablet formulation. While the environmental impact can be expressed in environmental Human Health burden due to resource use and emissions, the Human Health benefit of the pharmaceutical treatment of patients is currently not simultaneously taken into account. The study aims include a cradle-to-grave assessment of all Human Health impacts of the production, administration and disposal of two antipsychotics for the treatment of schizophrenia. This is complemented with the environmental impact of health care providers such as hospitals. The aim is to holistically quantify to what extent the environmental Human Health burden compares to the Human Health benefit associated with the treatment.

**Methods:**

We applied an overall framework which included Life Cycle Assessment to model the environmental Human Health impacts of the pharmaceutical supply chain, administration and disposal of the drug and health care providers. To model the patient benefit, this was complemented with a Markov model with a 1-year time horizon. Three patient groups were modeled: medicine coverage of paliperidone palmitate for either one month (PP1M) or three months (PP3M) at a time, and compared to Treatment Interruption (TI) as a control group. Outcomes were quantified using Years of Life Lost (YLL), Years Lived with Disability (YLD) and Disability-Adjusted Life Years (DALY).

**Results:**

The main environmental impacts were visits to the psychiatrist and psychiatric hospitals. The pharmaceutical supply chain had a limited impact. For 1000 patients for 1 year, PP1M and PP3M respectively avoided 0.38 and 0.49 environmental DALYs compared to TI. PP1M and PP3M further avoided 45.60 and 57.87 YLL and 23.31 and 29.91 YLD compared to TI. The main outcome was the sum of environmental DALYs, YLL and YLD, in which PP1M and PP3M respectively avoided 69.29 and 88.26 DALYs. Alternative analysis of Quality-Adjusted Life Years confirmed the results.

**Conclusions:**

The overall environmental burden was lower for PP1M and PP3M treatment than Treatment Interruption because patients are kept more stable, which reduces the environmental burden due to hospitals. Moreover, the Human Health burden was outweighed by the Human Health benefit.

**Electronic supplementary material:**

The online version of this article (10.1186/s12913-019-4247-2) contains supplementary material, which is available to authorized users.

## Background

The field of environmental impact assessment of pharmaceutical products has evolved in recent years, shifting its focus from the pharmaceutical supply chain to a complete health care pathway. This has been made explicit in guidance and policy documents by the Sustainable Development Unit (SDU) of the English National Health Service (NHS) [1].

The expansion of this scope should be reflected in the inclusion of health care providers such as hospitals next to the pharmaceutical supply chain, considering all resource use and emissions of hazardous compounds associated with care pathways [2–5]. In the field of Life Cycle Assessment (LCA), these resources and emissions can be linked to an environmental cause-effect chain, which finally results in damage to three main Areas of Protection (AoP): Natural Resources, Natural Environment and Human Health [6]. Traditionally LCA focuses on the burden of the products and services it analyses, with the benefit being defined as the products or services themselves. However, health care pathways represent a clear Human Health benefit to patients, which should be included in a holistic assessment and compared to the environmental Area of Protection Human Health burden. This is also recognized by the Swedish national pharmaceutical strategy [7].

From the perspective of health care professionals, it is now agreed that environmental criteria should be considered when making decisions on health care interventions, as confirmed by health care decision makers from key industrialized countries such as the US, Canada, UK and Germany [8]. However, the methodology to simultaneously capture both the environmental impact and the patient benefit of full health care pathways is currently missing.

We propose and evaluate a new approach and scope that allows the holistic quantification of the full burden and benefit of a health care pathway [9–11].

This demonstration study examines the treatment of schizophrenia, which is a devastating, long-term illness with a prevalence of around 0.7% worldwide [12]. The occurrence of an acute psychotic episode or relapse severely affects the quality of life and mortality of patients [13, 14]. Treatment with antipsychotics is recommended to manage psychotic symptoms and prevent relapse [15]. However, many patients show limited adherence and multiple longer periods of interrupted treatment, which are associated with worsening of symptoms and risk of relapse [16–21]. This is a well-established challenge for patients and families, which can be addressed by long-acting antipsychotic injections, ensuring medical coverage for a number of weeks or months [22, 23]. This study assesses the treatment effect of two long-acting antipsychotic injections: paliperidone palmitate once-monthly injection (PP1M) and paliperidone palmitate three-monthly injection (PP3M). NanoCrystal® technology is used to formulate the medicine suspension for both PP1M and PP3M, but due to an increased particle size PP3M has a longer sustained release of active ingredient [24–28].

The performance of PP1M and PP3M is quantified with a modified Cost-Effectiveness Analysis (CEA), as used by the pharmaceutical industry and health care policy decision makers [29]. This approach covers how a patient feels, functions and survives as a result of the pharmaceutical treatment [30]. Two metrics that can be used to express this effectiveness in patients’ quality and quantity of life are the Quality-Adjusted Life Years (QALY) and Disability-Adjusted Life Years (DALY) [31–35]. These patient outcomes are then compared to the environmental impact, which represents the ‘cost’ of the treatment.

This study aims to holistically quantify and compare the global environmental Human Health burden due to resource use and emissions and the patient Human Health benefit from treatment, with the patient consumption profile, calculated for each individual patient, as a functional unit.

## Methods

### Overall framework

**Fig. 1:**
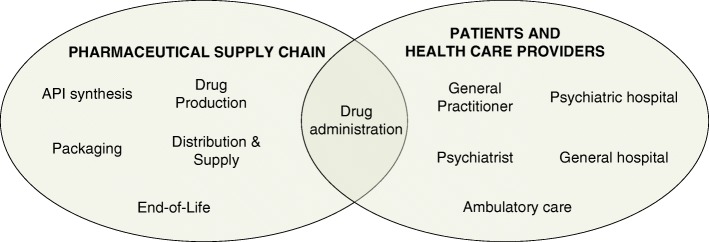
Drug administration is where the patient and the pharmaceutical supply chain overlap

Figure 1 gives an indicative overview of the framework applied in the study, with finer detail to follow. The environmental Human Health burden is quantified by accounting for all resource use and emissions from the relevant actors and phases in both the pharmaceutical supply chain and the health care providers. The Human Health benefit is located at the center of the figure, where the patient receives treatment.

**Fig. 2:**
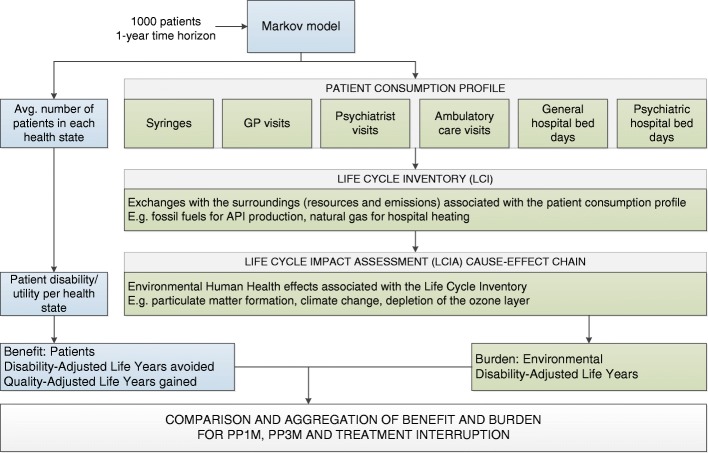
Interaction between Markov model and Life Cycle Assessment. Abbreviations: GP, general practitioner; PP1M, paliperidone palmitate once-monthly injection; PP3M, paliperidone palmitate three-monthly injection; TI, treatment interruption

The methodological framework is detailed in Fig. 2, which shows the interaction between the Markov model and the LCA. The Markov model determines the patient health benefit and provides the patient consumption profile. The Life Cycle Inventory (LCI) is then constructed followed by a Life Cycle Impact Assessment (LCIA) to determine the Human Health burden. Both the Human Health benefit and burden are then compared and aggregated.

### Part I: human health benefit

We define and compare three patient groups: treatment with paliperidone palmitate once-monthly injection (PP1M), treatment with paliperidone palmitate three-monthly injection (PP3M) and Treatment Interruption (TI).

Patients in Treatment Interruption are considered not to request medical treatment on their own initiative. However, once hospitalized for an unscheduled or unforeseen admission, these patients receive the same medical care as patients in the PP1M and PP3M treatment groups.

#### Model structure and design

We developed a Markov model to simulate disease outcomes of patients for PP1M and PP3M treatment, as well as Treatment Interruption. The model was built in Microsoft Excel. The population is a hypothetical Belgian patient cohort eligible for the maintenance treatment of schizophrenia [36–38]. In order to utilize evidence from the Randomized Clinical Trials (RCT) of paliperidone palmitate, we defined the age of the patients in the Markov model as 19–65 years, which matches the age of the patients in the RCTs [39–41]. Before starting PP3M, patients first require an initiation treatment on PP1M of four months. To align the model with the structure of the RCT, we assumed that this initiation was completed at the start of the model. The patients in PP1M and TI have also completed initiation on PP1M before the first model cycle.

The model envelops 5 health states. The ‘Stable: Adherent’ state represents patients that are not in relapse and are adherent to the medication. Patients in the ‘Stable: Non-adherent’ state are not in relapse but have completely discontinued their medication. Patients in relapse can be treated in a hospital or an ambulatory care setting in respectively the ‘Relapse: Hospitalization’ and ‘Relapse: Ambulatory care’ states. The ‘Death’ state is the absorbing state.

**Fig. 3:**
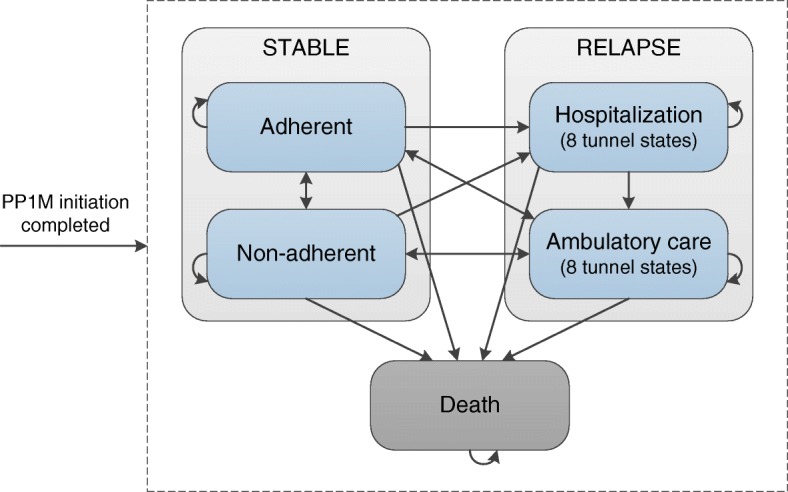
Markov model simulating the disease outcomes

Figure 3 displays the model states. Moving from relapse back to the stable states was found to be time-dependent. Therefore we introduced tunnel states with different transition probabilities dependent on the time that patient have stayed in the relapse state. A full description of the tunnel states can be found in Additional file 1 , page 2–3.

All the patients are considered adherent in the first cycle of the model. Patients in Treatment Interruption do not request medical treatment on their own initiative. Therefore, they are only adherent for the first model cycle as a result of the remaining medicine coverage. From the second cycle onwards patients can discontinue treatment, experience a relapse, die or return to one of the previous health states. Patients in the PP3M state can only discontinue treatment every 3 months. We defined relapse as an acute psychotic episode which can be treated in either a hospital or an ambulatory care setting. Many definitions of relapse of patients with schizophrenia are mentioned in the literature including patient functioning, events such as hospitalization and multiple symptom rating scales. However, there is no golden standard as yet [17, 42–44].

When patients recover from a relapse they return to either the Adherent or Non-adherent state. When patients return to the Stable: Adherent state they re-initiate PP1M or PP3M medication through the recommended re-initiation regimen.

We chose a time horizon of 1 year because of the patients’ tendency to switch to other treatments even within a 1-year timeframe [45]. In this study we assume that patients stay on or return to the same medication throughout the year. We adopted a cycle length of 1 month to match the medical coverage of PP1M.

#### Transition probabilities

We used data from the clinical trials of paliperidone palmitate and hospitals in Belgium. We carried out 9 literature reviews to further support the transition probabilities. Real-world evidence was used when available and secondary analysis was performed, for instance to isolate the patient group diagnosed with schizophrenia. The search strategies can be found in Additional file 1, page 6–15. An overview of the transition probabilities can be found in the Additional file 2: Tables A.5, A.6, A.7.

The monthly probability of relapse from the Stable: Adherent state was obtained from Savitz et al. for PP1M (0.0086) and for PP3M (0.0075) [41]. The monthly probability for relapse from the Stable: Non-adherent state was obtained from the placebo arm in Hough et al. for PP1M and TI (0.0639) and from the placebo arm in Berwaerts et al. for PP3M (0.0282) [39, 40]. The placebo arms in these studies were first stabilized on PP1M or PP3M and then randomized to placebo. The fact that patients on placebo are not fully discontinued is considered conservative, as fully discontinued patients would be worse off due to less contact with health care professionals. Analysis of the literature agrees with the used values, as detailed in Additional file 1, page 6–9.

According to a sub-set analysis of Lorant et al., 69% of patients in relapse are hospitalized in Belgium [46]. Consequently, 31% are in ambulatory care.

The length of relapse was assumed equal for patients who were hospitalized or in ambulatory care. For hospitalization it was assumed that patients were first brought to the general hospital following an acute relapse episode. After two days the patients are transferred to a psychiatric hospital for the remainder of the relapse (Audenaert K., personal communication). This is the case for general hospitals without a long-term psychiatric care unit. Two psychiatric hospitals provided recent data on the average length of an admission. Data from one hospital was sufficiently detailed for analysis. The graph of hospital discharge over time was right skewed, with outliers driving the mean to higher values. Most patients were discharged before the mean duration, with some patients staying for longer periods. Given this time-dependency of discharge, we introduced tunnel states in the model. The same length of stay in a psychiatric hospital was assumed for PP1M or PP3M treatment or Treatment Interruption.

After a relapse, patients have a 72% probability of becoming Stable: Adherent and a 28% probability of becoming Stable: Non-adherent [47].

We extracted the probability of treatment discontinuation from [48], based on real-world evidence from filled prescriptions at Belgian pharmacists. The monthly probability of discontinuation was 0.1200 for PP1M. Because no evidence exists for PP3M, we extrapolated the difference in discontinuation between the biweekly injection of Risperdal Consta and the monthly injection of PP1M. This led to a three-monthly discontinuation probability of 0.2816. The monthly probability of restarting the initial drug after discontinuation was 0.1489.

The probability of death was calculated from the general population mortality in Belgium [49]. We then multiplied this with the Standardized Mortality Ratio (SMR) for either stable schizophrenia or patients in relapse. The former was obtained from Saha et al. as 2.58, the latter from Hoang et al. as 6.2 [13, 50].

#### Disability sources and calculations

The disability of a disease can be weighted. A value of 0 represents no disability and 1 indicates full disability. We adopted disability weights from the World Health Organization (WHO) Global Burden of Disease (GBD) 2013 [51]. PP3M has a longer time between injections and hence a reduced injection burden. However, we did not assume a lower disability because of this. The disability weights did not include the occurrence of Adverse Events (AE) such as Extra Pyramidal Symptoms (EPS), weight gain or diabetes.

The disability weights can be found in Table 1. We calculated the Years Lived with Disability (YLD) as the disability weight of health state i (DW_(i)_) multiplied by the number of patients in health state i at month t (p_(i,t)_) [33].

$$ {YLD}_{\left(i,t\right)}={DW}_{(i)}\times {p}_{\left(i,t\right)} $$

**Table 1:** Disability weights and description of health states, available from Global Burden of Disease [51]

Health state	Disability	Health state description
Stable: Adherent	0.588	Schizophrenia: residual state Hears and sees things that are not real and has trouble communicating. The person can be forgetful, has difficulty with daily activities, and thinks about hurting himself (or herself).
Stable: Non-adherent	0.588	
Relapse: Hospitalization	0.778	Schizophrenia: acute state Hears and sees things that are not real and is afraid, confused, and sometimes violent. The person has great difficulty with communication and daily activities, and sometimes wants to harm or kill himself (or herself).
Relapse: Ambulatory care	0.778	
Death	1.000	

#### Years of life lost (YLL)

When a patient dies in the model the Years of Life Lost (YLL) are calculated as the number of deaths at age a and month t (N_(a,t)_) multiplied by the Years of Life Lost at age a (L_(a)_).

$$ {YLL}_{\left(a,t\right)}={N}_{\left(a,t\right)}\times {L}_{(a)} $$

The Years of Life Lost at death are 39.54, calculated from people aged 19–65 (weighted mean age: 41.61) in Belgium [49]. The fact that patients ‘age’ each month and have a lower potential YLL as the model progresses was taken into account. The YLL were not discounted [33].

The YLL are added to the YLD to calculate the Disability-Adjusted Life Years (DALY):

$$ DALY= YLD+ YLL $$

We chose DALYs as the main outcome metric of this study. Age-weighting or discounting of patient outcomes was not considered as we followed the WHO guidelines for the quantification of DALYs [33, 52]. We assessed Quality-Adjusted Life Years (QALY) based on utility values as a secondary outcome to validate the results.

#### Utility sources and calculations

Utility values represent the self-perceived wellbeing of a person on a scale from 0 to 1 [34]. We obtained utility values from Briggs et al. [53]. The study provides utility values elicited from interviews with both patients and laypersons. We chose to adopt the utility values from the layperson group, which complies with the Belgian guidelines and matches with the methodology of the GBD disability values that are also weighted by the general public [15, 52].

We complemented the utilities with the work of Osborne et al., which studies the difference in utility based on the time between injections in otherwise equal patients [54]. The outcome suggests a significantly higher utility for patients that are adherent on PP3M. Both Briggs and Osborne used the Time Trade-Off (TTO) method to elicit utilities. We included utility decrements for the following Adverse Events (AE): acute Extra Pyramidal Symptoms (EPS), weight gain (> 7% increase) and diabetes. Medication use can trigger AE, therefore the decrements were included in the Stable: Adherent state but also in the Stable: Non-adherent state. The decrements were weighted depending on the AE probability of occurrence in the Randomized Clinical Trials (RCT) [39–41].

The utility values can be found in Table 2. We calculated QALYs by multiplying the utility value of health state i with the time that population p spends in that health state (t_(i,p)_) [29, 55].

$$ {QALY}_{\left(i,p\right)}={Utility\ value}_{(i)}\times {t}_{\left(i,p\right)} $$

**Table 2:** Utility values of the health states [53, 54]

Health state	Utility	Source & calculation
PP1M		
Stable: Adherent	0.865	Layperson sample
Stable: Non-adherent	0.865	Assumed equal to Stable: Adherent
Relapse: Hospitalization	0.479	Layperson sample
Relapse: Ambulatory care	0.479	Layperson sample
Death	0.000	Assumed 0.000
PP3M		
Stable: Adherent	0.916	Layperson sample and added benefit for time between injections
Stable: Non-adherent	0.865	Assumed equal to Stable: Adherent
Relapse: Hospitalization	0.479	Layperson sample
Relapse: Ambulatory care	0.479	Layperson sample
Death	0.000	Assumed 0.000
Treatment Interruption		
Stable: Adherent	0.865	Layperson sample
Stable: Non-adherent	0.865	Assumed equal to Stable: Adherent
Relapse: Hospitalization	0.479	Layperson sample
Relapse: Ambulatory care	0.479	Layperson sample
Death	0.000	Assumed 0.000
Utility decrements for adverse events		
Acute EPS	0.291	Layperson sample (0.865–0.574)
Weight gain	0.086	Layperson sample (0.865–0.779)
Diabetes	0.153	Layperson sample (0.865–0.712)

Abbreviations: PP1M, paliperidone palmitate once-monthly injection; PP3M, paliperidone palmitate three-monthly injection; EPS, Extrapyramidal Symptoms

### Part II: human health burden

#### Goal and scope

The goal is to quantify and compare the environmental Human Health burden associated with PP1M or PP3M treatment and Treatment Interruption. The functional unit is the patient consumption profile, defined as the use of health care pathway elements in Belgium for 1 year for 1000 patients. This includes the number of used PP1M and PP3M syringes, visits to the GP and psychiatrist, ambulatory care visits and days spent in general and psychiatric hospitals. The monthly use of health care pathway elements per health state per patient is displayed in Table 3. Regardless of the frequency of antipsychotic injection, we consider three psychiatrist visits per month for all stable patients, as psychiatrists also provide general follow-up in addition to administering medication (Audenaert K., personal communication).

**Table 3:** The monthly use of health care pathway elements per health state per patient

Health state	Syringes	GP visits	Psychiatrist visits	Ambulatory care visits	General hospital bed days	Psychiatric hospital bed days
Stable: Adherent PP1M	1 PP1M syringe	0.3	3	0	0	0
Stable: Adherent PP3M	0.33 PP3M syringe	0.3	3	0	0	0
Stable: Non-adherent	0	0.3	3	0	0	0
Relapse: Ambulatory care	0	0	6.2	2.17	0	0
Relapse: Hospitalization month 1	0	0	0	0	2	28
Relapse: Hospitalization month 2-8^a^	0	0	0	0	0	30
Death	0	0	0	0	0	0

Abbreviations: PP1M, paliperidone palmitate once-monthly injection; PP3M, paliperidone palmitate three-monthly injection; GP, General Practitioner

^a^Patients can stay in the 8th hospitalization state for multiple cycles, see Additional file 1, page 2–3

We calculated the environmental impact of the health care pathway elements with the Life Cycle Assessment (LCA) methodology. This study focuses on the Human Health burden associated with environmental impacts.

The health care pathway elements are reflected in the scope of the LCA as previously shown in Fig 1. Both the pharmaceutical supply chain and the health care providers are considered. The former envelops the Active Pharmaceutical Ingredient (API) synthesis, drug production, packaging, distribution & supply and End-of-Life phases. The latter contains General Practitioner (GP) and psychiatrist, ambulatory care, psychiatric hospitals and general hospitals. The two fields overlap at the drug administration.

The LCA includes the use of chemicals, energy sources, transport, water, industrial waste treatment, packaging materials and End-of-Life disposal and fate of the drug. The cost of infrastructure does not allow clear allocation to the product, due to the uncertain lifetime of fixed equipment and buildings and is therefore not taken into account.

#### Methodology

The data and results in this study were obtained, processed and are presented according to the ISO 14040 and ISO 14044 series [56, 57] and International Reference Life Cycle Data System (ILCD) guidelines [58–60]. One exception is made for the Human Health endpoint indicator, as ISO 14044 does not support the grouping of midpoint impact categories into endpoints. The ILCD does support the use of endpoint indicators, although some of the midpoint indicators used to calculate this endpoint receive an interim recommendation [59]. The main part of the Human Health burden is attributable to Climate Change and Particulate Matter Formation which both receive the highest classification (classification I: recommended and satisfactory) on the midpoint level. On the endpoint level, Climate Change is proposed as the best among the analyzed methods while Particulate Matter Formation receives classification I/II (recommended and satisfactory/recommended but in need of some improvements). The Life Cycle Impact Assessment (LCIA) clearly details which characterization factors were used.

#### Life cycle inventory

The inventory for the pharmaceutical supply chain was gathered on-site at the Janssen Pharmaceutica sites in Cork (Ireland), Geel and Beerse (Belgium). The system boundaries of the foreground processes (the processes that were analyzed in detail) were the limits of the production plants. In addition, we also included the off-site industrial waste treatment operations such as distillation or incineration that were outsourced to third parties. Transport of the intermediate products between the Cork, Geel and Beerse sites was also taken into account. We included the basic unit operations as well as the main plant supporting processes. Primary data was used, all life cycle stages were included and the electricity mix was adapted depending on the origin of the electricity per production site.

Data for the API chemical synthesis of the active ingredient paliperidone palmitate and Drug Production of the PP1M and PP3M syringes was retrieved from Batch Production Reports, Cleaning Procedures, Equipment Manuals, yearly planning and partly through a shortcut LCA tool developed by Van der Vorst et al. specifically for the production plant in Geel [61]. The differences in NanoCrystal® formulation between PP1M and PP3M were included. The resource use of supporting processes such as heating, cooling and generation of purified water and steam was included. The (air) Heating, Ventilation and Air Conditioning (HVAC) system was included for the production in Cork and Beerse but not for Geel. This is a limitation as the LCA tool by Van der Vorst et al. does not include the HVAC system [61]. The industrial waste treatment of water-based and organic waste was taken into account.

As the PP1M and PP3M medicines come in different dosages, it was chosen to analyze the environmental burden of the dosage with the highest market share, which is 100 mg-eq. for PP1M and 350 mg-eq. for PP3M (Janssen Pharmaceutica, personal communication). The dosage in mg equivalents reflects the mass of the pharmacologically active compound paliperidone, where e.g. 100 and 350 mg-eq. relate to 156 mg and 546 mg paliperidone palmitate respectively.

The Packaging included the electricity use and HVAC of the packaging line and the primary, secondary and tertiary packaging materials. Packaging materials specific for the Belgian market were analyzed. Rejections by visual inspection of the syringe before packaging were included.

The Distribution & Supply included the transport from the production site in Beerse (Belgium) to the European distribution center in La Louvière (Belgium). This was added to the average distance from the distribution center to a Belgian psychiatrist (69.225 km), who administers the medicine in this model. The latter was calculated by the Geography department at Ghent University (Fransen K., personal communication). The yearly returns and destructions were included.

The End-of-Life phase considers paliperidone, the active metabolite of paliperidone palmitate. The total mass of paliperidone administered to the patient is subtracted by the percentage of API metabolized in the patient (41%) and removed in the WWTP (64%) (Janssen Pharmaceutica, personal communication). For the latter an approximation was used from Vergeynst et al. based on risperidone, which is identical to paliperidone except for one hydroxyl group [62].

The End-of-Life is based on Environmental Risk Assessment (ERA). When multiple measurements were available, e.g. for K_OC_ (the adsorption coefficient), the worst-case value was adopted. The End-of-Life assessment also included the waste disposal of the packaging materials and the hazardous waste disposal of the syringe.

Secondary data for the background processes (the processes that support the foreground) such as energy and chemicals were extracted from the ecoinvent v3.1 database using SimaPro v8 software [63].

The LCI of the health care providers consists of the average transport distance and the on-site energy and water use.

The average transport distance from a Belgian household to the closest three GP’s (1.091 km) or psychiatrists (7.055 km) and the closest hospital (10.933 km) was calculated by the Geography department at Ghent University (Fransen K., personal communication). The transport was assumed by car, as a modal split for health care-related transport was not readily available. The number of visits to the GP and psychiatrist was based on results from an expert panel of Belgian psychiatrists [64].

For the general and psychiatric hospitals we included the directly measured energy and water use based on yearly reporting figures, recalculated per bed day. The energy included electricity, natural gas and fuel. Any off-site generation of e.g. heat was not included. Data was obtained from 3 general hospitals and 5 psychiatric hospitals in the Flanders region. The general hospitals were the UZ Ghent hospital (Ghent), part of the UZ Leuven hospital (Leuven) and the AZ St. Lucas hospital (Ghent). The psychiatric hospitals were St. Camillus (Sint-Denijs-Westrem), PC Caritas (Melle), Dr. Guislain (Ghent), St. Jan (Eeklo) and Zoete Nood Gods (Lede). The amount of beds represented was 1981 for the general hospitals and 1071 for the psychiatric hospitals. A possible reduction in resource use at the home of the patient was not taken into account.

Patients in the Relapse: Ambulatory care state receive house visits from a nurse. In this case the nurses visit several patients in a row. Data from the mobile team at the Psychiatric Centre Gent-Sleidinge was used to calculate the average number of visits per month (2.17) and transport distance by car per patient (5.17 km). This is based on a total of 3018 house visits.

#### Life cycle impact assessment

The Impact Assessment focused on the impact categories with an effect on Human Health: Climate Change, Human Toxicity, Ionizing Radiation, Ozone Depletion, Particulate Matter Formation and Photochemical Oxidant Formation [65–69]. These were used to calculate the EndPoint Human Health burden through the ReCiPe v1.11 impact assessment method, which is identified as the best practice model for Human Health burden [6, 60, 70, 71].

The End-of-Life impact assessment of the molecule considered emissions to continental freshwater using the USEtox methodology [60, 72].

The results of the impact assessment were subdivided according to the type of resource or service: water, nitrogen, chemicals (reagents and solvents), energy (natural gas, electricity, fuel), packaging materials, industrial waste treatment, transport and End-of-Life.

#### Value choices

The Human Health damage can be calculated using different sets of Value Choices, each representing specific requirements for the discounting and considered time horizon of environmental effects. The Value choices also include the age-weighting of populations on which the environmental effects manifest [73, 74]. The consensus-driven Hierarchical perspective was chosen. This perspective considers a long time horizon for environmental effects to manifest and bases itself on scientific consensus, as opposed to the Individualist (optimistic) and Egalitarian (pessimistic) perspectives [59]. The use of a 0% discount factor and the avoidance of age-weighting is consistent with the assessment of disease outcomes resulting from treatment used in the Markov model. The applied time horizon for the Human Health damage is 100 years, which does not correspond to the 1-year time horizon used in the Markov model. However, matching time horizons in this case would not benefit the research. On the contrary, given that effects of compounds take years, if not decades to manifest in the environment it is opportune to choose two different time horizons: a short and manageable time horizon for the patient outcomes and longer time horizon to capture the environmental effects [6].

### Towards a net health effect

Both the patient health benefit and the environmental Human Health burden of a pharmaceutical treatment can be expressed in DALYs. Therefore we propose to merge these two outcomes into a single score. We calculate the ‘net health effect’ (DALY_net_) as:

$$ {DALY}_{patient}={YLD}_{patient}+{YLL}_{patient} $$

$$ {DALY}_{net}={DALY}_{patient}+{DALY}_{env} $$

With YLD_patient_ the disability of the 1000 patients, YLL_patient_ the loss of life years of the patients, DALY_patient_ the sum of the previous and DALY_env_ the environmental DALYs. The DALY_net_ is then calculated and compared across treatment with PP1M or PP3M and Treatment Interruption.

### Sensitivity analysis

A sensitivity analysis was performed as defined in Additional file 1, page 18–20. The sensitivity of the Markov model inputs with respect to the Human Health benefit was assessed. As the patient consumption profile is based on the same inputs, a range of environmental Human Health burden results was obtained at the same time.

## Results

The disease outcomes of the Markov model for 1000 patients during 1 year, as well as the environmental burden are listed in Table 4. TI, PP1M and PP3M scenarios yielded 626.80, 603.49 and 596.90 YLDs respectively, resulting in an YLD reduction of 23.31 (− 3.72%) and 29.91 (− 4.77%) for PP1M and PP3M respectively compared to TI.

**Table 4:** Patient consumption profile and Human Health benefit and burden results

1. Patient consumption profile	TI	PP1M	PP3M
Syringes (1 M)	–	8055	94
Syringes (3 M)	–	–	2564
GP visits	2883	3322	3447
Psychiatrist visits	34,109	35,224	35,528
Ambulatory care visits	1849	701	371
General hospital bed days	791	335	178
Psychiatric hospital bed days	43,864	16,406	8699
2. Human Health benefit			
Years Lived with Disability (YLD)			
Total patient YLDs	626.80	603.49	596.90
ΔYLDs of treatment vs. TI (%)	–	−23.31 (−3.72%)	−29.91 (−4.77%)
Years of Life Lost (YLL)			
Total patient YLLs	346.23	300.63	288.36
ΔYLL of treatment vs. TI (%)	–	−45.60 (−13.17%)	−57.87 (−16.71%)
Disability-Adjusted Life Years (DALY) = YLD + YLL			
Total patient DALYs	973.03	904.12	885.26
ΔDALY of treatment vs. TI (%)	–	−68.91 (−7.08%)	−87.77 (−9.02%)
Quality-Adjusted Life Years (QALY)			
Total patient QALYs	785.22	830.54	881.35
ΔQALY of treatment vs. TI (%)	–	+ 45.32 (+ 5.77%)	+ 96.13 (+ 12.24%)
3. Human Health burden			
Environmental DALYs			
Total environmental DALYs	0.954	0.579	0.468
ΔDALYs of treatment vs. TI (%)	–	−0.375 (−39.32%)	−0.487 (−51.00%)
4. Net Human Health effects (net DALYs = YLD + YLL + environmental DALYs)			
Total net DALYs	973.98	904.70	885.73
ΔDALY of treatments vs. TI	–	−69.29 (−7.11%)	−88.26 (−9.06%)

Abbreviations: PP1M, paliperidone palmitate once-monthly injection; PP3M, paliperidone palmitate three-monthly injection; TI, Treatment Interruption

The Years of Life Lost (YLL) were 346.23, 300.63 and 288.36 YLL for TI, PP1M and PP3M respectively, resulting in an YLL reduction of 45.60 (− 13.17%) and 57.87 (− 16.71%) for PP1M and PP3M respectively compared to TI.

Hence, TI, PP1M and PP3M resulted in 973.03, 904.12 and 885.26 DALYs respectively. Compared to TI, PP1M and PP3M avoided 68.91 (− 7.08%) and 87.77 (− 9.02%) DALYs respectively.

The alternative analysis yielded 785.22, 830.54 and 881.35 QALYs for TI, PP1M and PP3M, which resulted in a QALY gain of 45.32 (+ 5.77%) and 96.13 (+ 12.24%) QALYs for PP1M and PP3M respectively compared to TI.

Analysis of environmental Human Health burden resulted in 0.954, 0.579 and 0.468 environmental DALYs for TI, PP1M and PP3M respectively. Compared to TI, PP1M and PP3M avoided 0.375 (− 39.32%) and 0.487 (− 51.00%) environmental DALYs respectively.

The net DALY burden including both patient and environmental outcomes amounted to 973.98, 904.70 and 885.73 DALYs for Treatment Interruption or treatment with PP1M or PP3M respectively, which resulted in a DALY reduction of 69.29 (− 7.11%) and 88.26 (− 9.06%) for PP1M and PP3M respectively compared to TI.

**Fig. 4:**
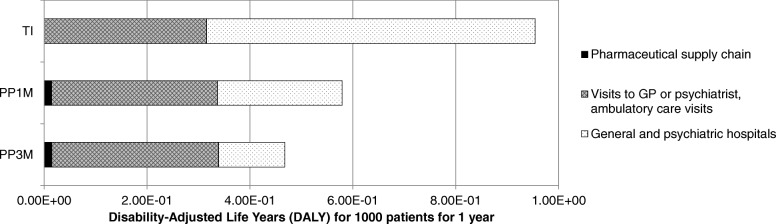
Environmental Human Health burden in Disability-Adjusted Life Years (DALYs): Abbreviations: GP, General Practitioner; PP1M, paliperidone palmitate once-monthly injection; PP3M, paliperidone palmitate three-monthly injection; TI, Treatment Interruption

The environmental Human Health burden was divided in three main parts shown in Fig. 4: the pharmaceutical supply chain, visits to the GP or psychiatrist as well as ambulatory care visits and general and psychiatric hospitals. Table 5 provides more detailed results with a subdivision concerning the resource type. The difference between industrial waste treatment and End-of-Life is that the former concerns treatment of waste from pharmaceutical production sites, whereas the latter envelops post-consumer waste. The negative values in the table were caused by waste incineration processes with energy recovery. This avoided the use of virgin resources and was accounted as an environmental gain. A table with a subdivision on midpoint categories can be found in Additional file 1, page 16–17.

**Table 5:** Environmental Human Health burden in Disability-Adjusted Life Years (DALYs)

TI	API	Drug Production	Packaging	Distribution & Supply	End-of-Life disposal & drug fate	GP visits	Psychiatrist visits	Ambulatory care visits	General hospital days	Psychiatric hospital days
Water	N/A	N/A	N/A	N/A	N/A	0.00E+ 00	0.00E+ 00	0.00E+ 00	2.20E-04	3.99E-03
Nitrogen	N/A	N/A	N/A	N/A	N/A	0.00E+ 00	0.00E+ 00	0.00E+ 00	0.00E+ 00	0.00E+ 00
Chemicals - Reagents	N/A	N/A	N/A	N/A	N/A	0.00E+ 00	0.00E+ 00	0.00E+ 00	0.00E+ 00	0.00E+ 00
Chemicals - Solvents	N/A	N/A	N/A	N/A	N/A	0.00E+ 00	0.00E+ 00	0.00E+ 00	0.00E+ 00	0.00E+ 00
Energy - Natural Gas	N/A	N/A	N/A	N/A	N/A	0.00E+ 00	0.00E+ 00	0.00E+ 00	7.20E-04	4.29E-02
Energy - Electricity	N/A	N/A	N/A	N/A	N/A	0.00E+ 00	0.00E+ 00	0.00E+ 00	3.85E-02	3.79E-01
Energy - Fuel	N/A	N/A	N/A	N/A	N/A	0.00E+ 00	0.00E+ 00	0.00E+ 00	2.73E-02	5.41E-02
Packaging materials	N/A	N/A	N/A	N/A	N/A	0.00E+ 00	0.00E+ 00	0.00E+ 00	0.00E+ 00	0.00E+ 00
Industrial waste treatment	N/A	N/A	N/A	N/A	N/A	0.00E+ 00	0.00E+ 00	0.00E+ 00	0.00E+ 00	0.00E+ 00
Transport	N/A	N/A	N/A	N/A	N/A	3.99E-03	3.06E-01	6.08E-03	5.49E-03	8.70E-02
End-of-Life	N/A	N/A	N/A	N/A	N/A	0.00E+ 00	0.00E+ 00	0.00E+ 00	0.00E+ 00	0.00E+ 00
PP1M										
Water	1.72E-06	4.38E-07	0.00E+ 00	0.00E+ 00	0.00E+ 00	0.00E+ 00	0.00E+ 00	0.00E+ 00	9.32E-05	1.49E-03
Nitrogen	6.25E-05	0.00E+ 00	0.00E+ 00	0.00E+ 00	0.00E+ 00	0.00E+ 00	0.00E+ 00	0.00E+ 00	0.00E+ 00	0.00E+ 00
Chemicals - Reagents	1.41E-03	1.89E-05	0.00E+ 00	0.00E+ 00	0.00E+ 00	0.00E+ 00	0.00E+ 00	0.00E+ 00	0.00E+ 00	0.00E+ 00
Chemicals - Solvents	5.94E-04	6.76E-08	0.00E+ 00	0.00E+ 00	0.00E+ 00	0.00E+ 00	0.00E+ 00	0.00E+ 00	0.00E+ 00	0.00E+ 00
Energy - Natural Gas	2.43E-03	2.06E-03	8.96E-04	0.00E+ 00	0.00E+ 00	0.00E+ 00	0.00E+ 00	0.00E+ 00	3.05E-04	1.60E-02
Energy - Electricity	2.74E-03	1.48E-04	7.95E-05	−7.29E-06	−4.02E-04	0.00E+ 00	0.00E+ 00	0.00E+ 00	1.63E-02	1.42E-01
Energy - Fuel	0.00E+ 00	0.00E+ 00	0.00E+ 00	0.00E+ 00	0.00E+ 00	0.00E+ 00	0.00E+ 00	0.00E+ 00	1.16E-02	2.02E-02
Packaging materials	ND	4.36E-08	2.65E-03	0.00E+ 00	0.00E+ 00	0.00E+ 00	0.00E+ 00	0.00E+ 00	0.00E+ 00	0.00E+ 00
Industrial waste treatment	9.95E-04	5.12E-07	0.00E+ 00	6.01E-06	0.00E+ 00	0.00E+ 00	0.00E+ 00	0.00E+ 00	0.00E+ 00	0.00E+ 00
Transport	1.61E-05	0.00E+ 00	1.16E-04	5.13E-05	0.00E+ 00	4.60E-03	3.16E-01	2.30E-03	2.33E-03	3.25E-02
End-of-Life	ND	ND	0.00E+ 00	0.00E+ 00	3.75E-04	0.00E+ 00	0.00E+ 00	0.00E+ 00	0.00E+ 00	0.00E+ 00
PP3M										
Water	2.19E-06	5.67E-07	0.00E+ 00	0.00E+ 00	0.00E+ 00	0.00E+ 00	0.00E+ 00	0.00E+ 00	4.95E-05	7.92E-04
Nitrogen	7.94E-05	0.00E+ 00	0.00E+ 00	0.00E+ 00	0.00E+ 00	0.00E+ 00	0.00E+ 00	0.00E+ 00	0.00E+ 00	0.00E+ 00
Chemicals - Reagents	1.79E-03	2.28E-05	0.00E+ 00	0.00E+ 00	0.00E+ 00	0.00E+ 00	0.00E+ 00	0.00E+ 00	0.00E+ 00	0.00E+ 00
Chemicals - Solvents	7.55E-04	8.59E-08	0.00E+ 00	0.00E+ 00	0.00E+ 00	0.00E+ 00	0.00E+ 00	0.00E+ 00	0.00E+ 00	0.00E+ 00
Energy - Natural Gas	3.09E-03	2.62E-03	3.33E-04	0.00E+ 00	0.00E+ 00	0.00E+ 00	0.00E+ 00	0.00E+ 00	1.62E-04	8.51E-03
Energy - Electricity	3.48E-03	1.88E-04	2.98E-05	−3.18E-06	−1.45E-04	0.00E+ 00	0.00E+ 00	0.00E+ 00	8.66E-03	7.51E-02
Energy - Fuel	0.00E+ 00	0.00E+ 00	0.00E+ 00	0.00E+ 00	0.00E+ 00	0.00E+ 00	0.00E+ 00	0.00E+ 00	6.14E-03	1.07E-02
Packaging materials	ND	7.57E-08	1.10E-03	0.00E+ 00	0.00E+ 00	0.00E+ 00	0.00E+ 00	0.00E+ 00	0.00E+ 00	0.00E+ 00
Industrial waste treatment	1.26E-03	6.51E-07	0.00E+ 00	2.62E-06	0.00E+ 00	0.00E+ 00	0.00E+ 00	0.00E+ 00	0.00E+ 00	0.00E+ 00
Transport	2.04E-05	0.00E+ 00	4.65E-05	2.24E-05	0.00E+ 00	4.77E-03	3.18E-01	1.22E-03	1.24E-03	1.73E-02
End-of-Life	ND	ND	0.00E+ 00	0.00E+ 00	1.48E-04	0.00E+ 00	0.00E+ 00	0.00E+ 00	0.00E+ 00	0.00E+ 00

Abbreviations: PP1M, paliperidone palmitate once-monthly injection; PP3M, paliperidone palmitate three-monthly injection; TI, Treatment Interruption; API, Active Pharmaceutical Ingredient; GP, General Practitioner; ND, Non-Determined; N/A, Not Applicable

For PP1M 54.48% of the environmental DALYs originated from psychiatrist visits, 36.59% from psychiatric hospital stays, 5.28% from general hospital stays and 2.46% from the pharmaceutical supply chain. For PP3M 68.04% resulted from psychiatrist visits, 24.03% from psychiatric hospital stays, 3.18% from the pharmaceutical supply chain and 3.47% from general hospital stays. For TI 59.36% was caused by psychiatric hospital stays, 32.01% from psychiatrist visits and 7.57% by general hospitals stays.

Visits to the psychiatrist and hospitalization in psychiatric hospitals caused the bulk of the Human Health burden. When looking at the underlying resource use for psychiatric hospitals, 78.27% of the total Human Health burden was due to electricity use, 11.19% due to fuel consumption and 8.87% due to natural gas use. Hospitalization in general hospitals represented only 2 days per full hospitalization, and was therefore responsible for a large DALY/day contribution. For the general hospitals 57.66% of the total burden was due to electricity use and 40.93% due to fuel consumption. Consequently, car transport and electricity use in hospitals were the main cause of Human Health burden.

Ambulatory care performed significantly better than hospitalization, contributing 0.40, 0.26 and 0.64% of the total environmental DALYs for PP1M, PP3M and TI respectively. The End-of-Life phase, a part of the supply chain with traditionally a high focus on environmental concerns had a negligible contribution to the total Human Health burden.

The results of the one-way and probabilistic sensitivity analysis can be found in Additional file 1, page 18–20. Overall, the model was robust in the sense that the conclusion did not change in any of the analysis.

## Discussion

The environmental Human Health burden is reduced for PP1M and PP3M compared to TI because of a decrease in hospitalization. Within the pharmaceutical supply chain the largest environmental impact is associated with the API synthesis. However, the health care providers together represent an impact 40 and 30 times larger than the pharmaceutical supply chain of PP1M and PP3M, respectively. Visits to the psychiatrist and psychiatric hospitals were the main hotspots. This reinforces the need for holistic assessments when analyzing the environmental performance of health care pathways.

Maintenance treatment of schizophrenia with PP1M and PP3M also leads to less patient DALYs than TI because the treatments prevent relapse. There is a striking difference in order of magnitude between the avoided patient DALYs and environmental DALYs: respectively 184 and 180 times more patient DALYs than environmental DALYs were avoided for PP1M and PP3M versus TI.

This is one of the first attempts to holistically quantify the Human Health benefit and burden of a full health care pathway. We expanded the environmental impact assessment of a health care pathway to include both the pharmaceutical supply chain and health care providers, for which multiple primary data sources were used. Methodologies from different fields of research were combined in a new quantitative approach, using a common metric for the Human Health performance of health care pathways.

The limitations of the study that should be noted are the following. Even though real-world evidence was used when available, the results of the Markov model are influenced by the Randomized Clinical Trials (RCT) that provided input data. Hence, the results of the Markov model should not be considered as real-world patient benefits or burdens. The one-way sensitivity analysis in Additional file 1, page 18–19 shows that the duration of relapse is the most sensitive parameter. Data availability is a limitation here, as this calculation is based on data from one hospital. The model was robust and not particularly sensitive to other parameters. The conclusion did not change in any of the sensitivity analysis. It should however be noted that the sensitivity analysis was based on point estimates, which were varied by the standard deviation, or ± 20% if the former was not available.

The results for both patient YLDs and QALYs are high. This may seem contradictory, as a high number of YLDs should be associated with a low number of QALYs. This can be partly explained by the origin of the disability weights and utility values. The Global Burden of Disease (GBD) includes self-harm as part of the disability description of both stable schizophrenia and relapse, which may partly explain the high disability rating [51]. Furthermore, the health state description of the GBD details severe symptoms, even for the residual or stable state. We consider this to be a conservative approach. The descriptions in Briggs et al. are less severe and do not include self-harm [53].

Both YLDs and QALYs could have been used as the main reporting metric on treatment impact on Human Health. In this case, we preferred YLDs for two reasons. First, the delta disability between stable schizophrenia and relapse is smaller, resulting in a more conservative benefit of avoided YLDs for treatment with PP1M or PP3M compared to TI. There is also no additional benefit for PP3M in the Stable: Adherent state. This can be considered a conservative approach. Second, the use of DALYs as a patient outcome matches the use of DALYs as an environmental Human Health burden metric in Life Cycle Assessment (LCA). This enables a direct comparison between both fields of research.

The LCA also has limitations. We only considered car transport for visits to the GP or psychiatrist. For the general and psychiatric hospitals we included energy sources and water but not the food, cleaning or other procurement. When patients relapse, health care providers administer emergency medication to suppress the symptoms. This medication was not included in the assessment, which is considered a conservative approach. The negligible environmental impact of the End-of-Life phase could be questioned. We chose a consensus model out of the multiple toxicity models that are available [72]. There is also a large spread in Absorption, Distribution, Metabolism, Excretion (ADME) and toxicity properties of drug substances [3, 75]. The small environmental impact could also be explained by the low dose regimen of the medicine. Therefore, the contribution of End-of-Life to the total impact in this case is not representative for all pharmaceuticals, although it does indicate that a broader focus may be warranted when considering the impact of pharmaceuticals in the environment.

There is an ongoing discussion on the grouping of midpoint categories to calculate endpoints in Life Cycle Assessment. We argue that it is justified in this case because of the opportunity to make a direct comparison between the Human Health benefit and burden [76]. Midpoint indicators are not suited to reflect this burden and only consider effects. However, we acknowledge the criticism of endpoint modeling for its high uncertainty [77].

We identified one prior study reporting patient and environmental outcomes of a pharmaceutical treatment [78]. For the treatment of type 2 diabetes, patient outcomes are reported in QALYs and the environmental assessment is in kg CO_2_ emissions. The carbon intensity of the treatment is obtained through a cost-based top-down approach of the average carbon footprint of pharmaceutical products procured by the NHS. While offering a transparent and simple method to include environmental impacts in health economic analysis, the results are not directly comparable to the outcomes in this study.

The English National Health Service (NHS) Sustainable Development Unit (SDU) reports that in 2015 pharmaceuticals were responsible for 11% of the total Carbon Footprint of the NHS [79]. The energy use of buildings is associated with 18% of the Carbon Footprint and transport causes 13% of the impact. The SDU uses a different environmental impact method: the Carbon Footprint. However, it is closely linked to Human Health damage, for which Climate Change is typically the main driver.

The results of the SDU suggest a higher contribution of pharmaceuticals and a lower contribution of buildings and transport than reported in this study. This could be attributed to the difference in scope. The SDU considers the full health care system while this study focuses on one disease area. The analysis was also performed in a different country. Transport and the degree and type of hospitalization are specific for each disease area and country. Furthermore, the drug dose per day in this study is low.

There is also a difference in approach. The care pathway modules defined by the SDU are similar to the health care pathway elements used in this study [1]. However, the SDU uses a top-down approach which enables a fast assessment of a full health care system. The current study uses a data intensive, but more detailed bottom-up approach that also includes the benefits at the service level.

The results of this study are specific for the treatment of patients with schizophrenia with long-acting antipsychotic injections in the Flanders region of Belgium. The findings suggest that treatment with PP1M and PP3M avoids Human Health burden for both patients and the environment. Three suggestions are proposed to enlarge this benefit even more.

First, ambulatory care could be promoted over hospitalization. If this is feasible from a treatment perspective, it would reduce the environmental Human Health burden considerably. Second, hospitals could reduce the impact of their electricity consumption by opting for a cleaner and more renewable energy mix. Third, environmentally sustainable transport in health care could be promoted. This is probably already the case, as we assumed that all transport is by car, where in reality patients might use public transport.

Our data suggest that academics and policymakers evaluating a pharmaceutical treatment should consider the full health care pathway including all environmental and patient Human Health impacts.

The outcomes of this study should be further tested and validated with research on other disease areas, countries, health care settings and standards. It is unlikely that the pharmaceutical treatment of patients will avoid environmental Human Health burden in all disease areas. For instance, the results of this study could be compared to a health care pathway with less intensive contact with health care providers and a higher daily dose of medication. This could provide further insights in the relationship between the health care providers and the pharmaceutical supply chain with respect to the ranges of Human Health burden.

The environmental part of this study only considers impacts on Human Health. Other Areas of Protection (AoP), such as depletion of natural resources and damage to the natural environment may be considered for inclusion in future studies to capture all environmental aspects.

## Conclusions

We consider the treatment of patients with schizophrenia in Belgium with paliperidone palmitate once-monthly or paliperidone palmitate three-monthly long-acting injections. The treatments are compared with Treatment Interruption and reduce the cradle-to-grave environmental Human Health burden, quantified as Disability-Adjusted Life Years (DALYs), because of a lower risk of relapse, leading to a reduction of hospitalization. Apart from hospitalization, car transport of patients represents the largest environmental Human Health burden. The treatments represent a clear Human Health benefit for the patients quantified as avoided DALYs, which is compared to the avoided burden using a common metric. The results of this demonstration study can help policymakers to identify and address the environmental Human Health hot spots of the treatment of schizophrenia in Belgium.

The patient health benefit in avoided DALYs is several orders of magnitude larger than the global environmental DALYs. Additionally, the environmental Human Health burden is reduced by treatment because of a lower risk of relapse, which reduces hospitalizations. This benefit versus benefit conclusion is opposed to the benefit versus burden result that one would expect [9].

While this study provides a first insight in the holistic quantification of the Human Health benefit and burden of a full health care pathway, these findings should be validated and contrasted with research based on real-world data in different disease areas and in multiple countries.

## **Additional files**

Additional file 1: Background information on model inputs, assumptions and limitations, literature reviews, Life Cycle Assessment results by midpoint category and sensitivity analysis. (DOCX 4880 kb)

Additional file 2: Transition probability matrices for the Markov model. (XLSX 14 kb)

## Data Availability

All data generated or analyzed during this study for the Markov model are included in this published article and its supplementary information files. The data that support the findings of the Life Cycle Assessment of this study are available from Janssen Pharmaceutica but restrictions apply to the availability of these data, which were used under license for the current study, and so are not publicly available. Data are however available from the authors upon reasonable request and with permission of Janssen Pharmaceutica.

## Abbreviations

AoP: Area of Protection
CEA: Cost-Effectiveness Analysis
DALY: Disability-Adjusted Life Years
LCA: Life Cycle Assessment
LCI: Life Cycle Inventory
LCIA: Life Cycle Impact Assessment
NHS SDU: National Health Service Sustainable Development Unit
PP1M: Paliperidone palmitate one-monthly injection
PP3M: Paliperidone palmitate three-monthly injection
QALY: Quality-Adjusted Life Years
TI: Treatment Interruption
YLD: Years Lived with Disability
YLL: Years of Life Lost
