# Supplementary material for: Human health benefit and burden of the schizophrenia health care pathway in Belgium: paliperidone palmitate long-acting injections
Source: BMC Health Serv Res. 2019 Jun 19;19:393. doi: 10.1186/s12913-019-4247-2 (PMC6585029; doi:10.1186/s12913-019-4247-2)
Supplement: Supplementary file 1 — Background information on model inputs, assumptions and limitations, literature reviews, Life Cycle Assessment results by midpoint category and sensitivity analysis. (DOCX 4880 kb) [file 12913_2019_4247_MOESM1_ESM.docx]

**Additional file**

Human Health Benefit and Burden of the Schizophrenia Health Care Pathway in Belgium: Paliperidone Palmitate Long-Acting Injections

Sam Debaveye^1^, Delphine De Smedt^2^, Bert Heirman^3^, Shane Kavanagh^4^, Jo Dewulf^1^

*^1^ Research Group Environmental Organic Chemistry and Technology (EnVOC), Faculty of Bioscience Engineering, Ghent University, Campus Coupure, Coupure Links 653, B-9000 Ghent, Belgium*

*^2^ Department of Public Health, Ghent University, Campus UZ, De Pintelaan 185, B-9000 Ghent, Belgium*

*^3^ Johnson & Johnson EHS&S, Janssen Pharmaceutica NV, Turnhoutseweg 30, B-2340 Beerse, Belgium*

*^4^ Health Economics, Janssen Pharmaceutica NV, Turnhoutseweg 30, B-2340 Beerse, Belgium*

Table of contents

[1. Model structure 2](#_Toc10567369)

[2. Overview of Markov model inputs, assumptions and limitations 4](#_Toc10567370)

[3. Literature reviews to support transition probabilities 6](#_Toc10567371)

[3.1 Relapse probability for TI – Randomized Controlled Trials 6](#_Toc10567372)

[3.2 Relapse probability for TI – fully untreated patients 8](#_Toc10567373)

[3.3 Relapse probability for TI – real-world evidence 9](#_Toc10567374)

[3.4 Probability of hospitalization or ambulatory care in relapse 10](#_Toc10567375)

[3.5 Probability of discontinuation for long-acting injections 11](#_Toc10567376)

[3.6 Length of hospitalization in Belgium 12](#_Toc10567377)

[3.7 Length of relapse in hospital and ambulatory care 13](#_Toc10567378)

[3.8 Length of relapse and share of hospitalization 14](#_Toc10567379)

[3.9 Probability of death 15](#_Toc10567380)

[4. Life Cycle Assessment by Human Health midpoint category 16](#_Toc10567381)

[5. One-way sensitivity analysis 18](#_Toc10567382)

[6. Probabilistic sensitivity analysis 20](#_Toc10567383)

[7. References 21](#_Toc10567384)

# **Model structure**

The length of a relapse in Relapse: Hospitalization was obtained from a psychiatric hospital, where the time between admission and discharge for an acute psychotic episode of schizophrenic patients was recorded. The mathematical distribution of this duration of hospitalization asked for time-dependent probabilities in terms of being discharged from the hospital. Therefore, tunnel-states were introduced for 8 consecutive months of hospitalization. This introduced a form of ‘memory’ in the Markov model. From the 8^th^ month the probability of discharge remains constant, therefore patients can stay multiple cycles in the 8^th^ tunnel state. At each hospitalization state, a patient could migrate to the next hospitalization state or could be discharged from the hospital. After discharge, the patient spends one additional month in Relapse: Ambulatory care for intensive follow-up. This reflects the practice in Belgium, where patients receive 4 weeks of additional ambulatory care follow-up after discharge (Audenaert K, personal communication). Patients that are in Relapse: Ambulatory care from the start have the same probabilities of returning to the stable state as the Relapse: Hospitalization patients have of being discharged from the hospital.

The visual overview of the Markov model health states is provided in Figure A.1.


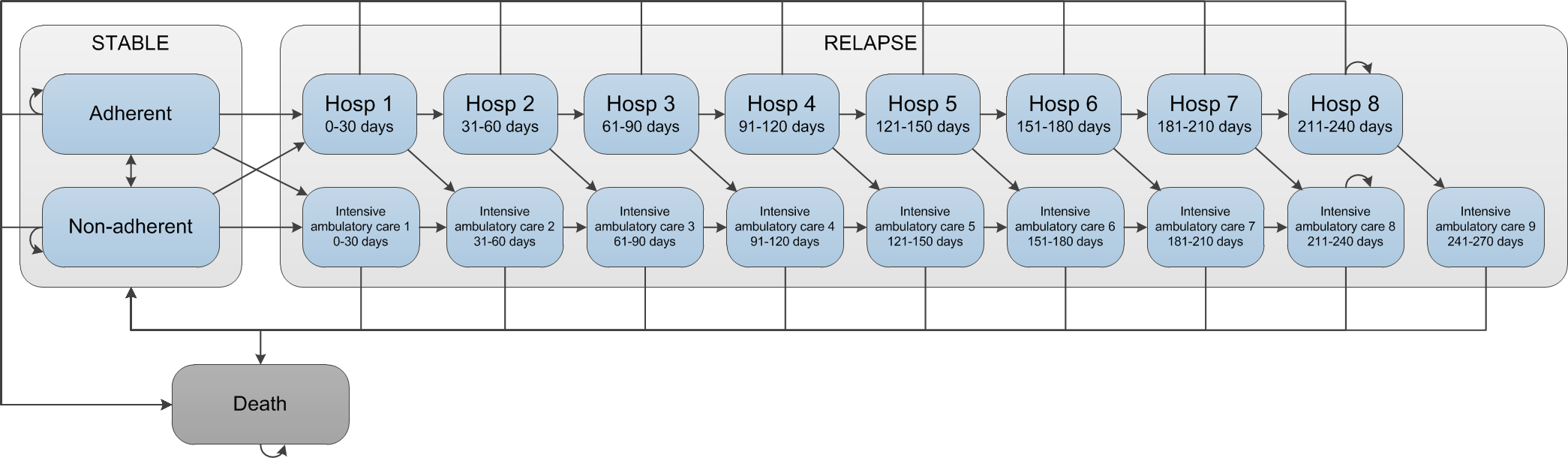


Figure A.1: The Markov model health states and transition arrows. **The tunnel states are included to reflect time-dependent parameters. When patients experience a relapse, the probability of discharge from the hospital is time-dependent. Therefore 8 consecutive hospitalization states were modeled. At each hospitalization state, a patient could migrate to the next hospitalization state or could be discharged from the hospital. After discharge, the patient spends one additional month in Relapse: Ambulatory care for intensive follow-up.**

# Overview of Markov model inputs, assumptions and limitations

An overview of the Markov model inputs, assumptions and limitations can be found in Tables A.1, A.2 and A.3.

Table A.1: Overview of main Markov model inputs.

| Model input | Source |
| --- | --- |
| Monthly probability of relapse from Stable: Adherent | [Savitz et al. (2016)](#_ENREF_13) |
| Monthly probability of relapse from Stable: Non-adherent | For PP1M: [Hough et al. (2010)](#_ENREF_7)  For PP3M: [Berwaerts et al. (2015)](#_ENREF_1) |
| Fraction of patients hospitalized or in ambulatory care during relapse | [Lorant et al. (2016)](#_ENREF_8), secondary analysis performed on patients diagnosed with schizophrenia |
| Length of relapse | Primary data from a psychiatric hospital in Flanders |
| Probability of becoming Stable: Adherent or Stable: Non-adherent after relapse | [Marcus et al. (2015)](#_ENREF_9) |
| Probability of treatment discontinuation | [Decuypere et al. (2017)](#_ENREF_5) |
| Probability of death: stable | Belgium general population mortality ([Statbel 2015](#_ENREF_14)) & [Saha et al. (2007)](#_ENREF_11) |
| Probability of death: relapse | Belgium general population mortality ([Statbel 2015](#_ENREF_14)) & [Hoang et al. (2011)](#_ENREF_6) |
| Disability weights | [Salomon et al. (2015)](#_ENREF_12) |
| Years of Life Lost at death | Belgium age composition tables ([Statbel 2015](#_ENREF_14)) |
| Utility values | [Briggs et al. (2008)](#_ENREF_3) (layperson group) and [Osborne et al. (2012)](#_ENREF_10) (influence time between injections) |

Table A.2: Overview of main study assumptions.

| Assumptions | Implication |
| --- | --- |
| For all patients: PP1M initiation of four months completed. | All patients have the same treatment history at the start of the model |
| All patients are considered adherent in first cycle of model | Conservative, as the Treatment Interruption group is in the Stable: Adherent state for one month |
| Treatment Interruption group does not request medical treatment on their own initiative | Conservative as RCT inputs for this group are based on patients on placebo, who are not fully discontinued |
| The Treatment Interruption group does receive the same medical care as PP1M and PP3M groups while hospitalized | Conservative, as recovering from relapse without medication would increase the benefit of the treatment groups compared to Treatment Interruption |
| Moving from relapse back to stable state is time dependent: use of tunnel states | Model reflects actual real-world data |
| Patients in PP3M group can only discontinue treatment every three months | Discontinuation is postponed. While not conservative, it should reflect the real-world situation. |
| Probability of treatment discontinuation for PP3M extrapolated from difference between Risperdal Consta and PP1M | Conservative estimate, as we extrapolated the difference between a 2-weekly vs. 4-weekly injection (factor 2 time difference) to a 4-weekly vs. 12-weekly injection (factor 3 time difference), without accounting for the 3/2 factor. |
| Length of relapse equal for patients who were hospitalized or in ambulatory care | Model results could change if different length of relapse was used for hospitalization and ambulatory care. The conclusion would remain the same as equal probabilities are used for PP1M, PP3M and Treatment Interruption. |
| Patients that are hospitalized are first brought to a general hospital and transferred to a psychiatric hospital after two days | Only impacts LCA results |

Table A.3: Overview of main study limitations.

| Limitations | Implication |
| --- | --- |
| Model results influenced by data from Randomized Clinical Trials | Model results should not be extrapolated as real-world results |
| Patients who recover from relapse and move to Stable: Adherent re-initiate the medicine they were on before relapse | Study does not consider medication other than PP1M and PP3M |
| Length of relapse based on data from one psychiatric hospital | Primary data from the hospital led to more conservative model results than literature sources on length of relapse in Belgium. Model results could change if detailed data from another psychiatric hospital was used. The conclusion would remain the same as equal probabilities are used for PP1M, PP3M and Treatment Interruption. |

# Literature reviews to support transition probabilities

## Relapse probability for TI – Randomized Controlled Trials

Search terms [title/abstract]: Schizophren* AND placebo AND relaps*

Databases: PubMed, Cochrane Reviews, TRIP Database, Epistemonikos, Web of Science

Date searched: 12/04/2016 – 03/05/2016

Dates searched:

Web of Science, PubMed: 2013-2016

Cochrane reviews, TRIP Database, Epistemonikos: 2006-2016

Conclusion: use the relapse probability of placebo arm from [Hough et al. (2010)](#_ENREF_7), which is supported by the literature

Multiple meta-analysis’ were performed on the 17 records extracted from literature using R v3.3.0 and the ‘metafor’ package. The Raw Incidence Rate (IR) was calculated through the rma.uni function, using a random effects model. As the most representative sub-analysis we considered the six studies where relapse also includes hospitalisation. The observed relapse probability of 0.556 with a 95% Confidence Interval of [0.395;0.717] was very similar to that of the placebo arm of Hough et al., 2010: 0.552 [0.450;0.655]. Therefore we chose to adopt the latter as the relapse probability is slightly lower (more conservative) while also supported by the literature.

PRISMA diagram for the search is displayed in Figure A.2.

Figure A.2: PRISMA diagram of the Pragmatic Literature Review of the relapse probability of placebo patients.

## Relapse probability for TI – fully untreated patients

Search terms: schizophren*:ti,ab AND relaps*:ti,ab AND (untreated:ti,ab OR nontreated:ti,ab OR 'non treated':ti,ab OR non-persisten*:ti,ab)

Databases: Embase

Date searched: 28/11/2016

Dates searched: All

Conclusion: use the relapse probability of placebo arm from [Hough et al. (2010)](#_ENREF_7), which is supported by the literature

PRISMA diagram for the search is displayed in Figure A.3.

Figure A.3: PRISMA diagram of the Pragmatic Literature Review on the relapse probability of fully-untreated patients.

## Relapse probability for TI – real-world evidence

Search terms: schizophren*:ti AND relaps*:ti AND ('longitudinal study'/exp OR 'prospective study'/exp OR 'retrospective study'/exp OR 'observational study'/exp OR 'medical record'/exp OR 'personal experience'/exp OR 'population'/exp OR 'cohort analysis'/exp OR naturalist*:ti,ab OR real*world:ti,ab OR regist*:ti,ab OR evidenc*:ti,ab)

Databases: Embase

Date searched: 09/11/2016

Dates searched: 2006-2016

Conclusion: use the relapse probability of placebo arm from [Hough et al. (2010)](#_ENREF_7), which is supported by the literature

PRISMA diagram for the search is displayed in Figure A.4.

Figure A.4: PRISMA diagram of the Pragmatic Literature Review on the real-world evidence of the relapse probability of discontinued or non-adherent patients.

## Probability of hospitalization or ambulatory care in relapse

Search terms [title/abstract]: (schizophren*) AND ((commun* care) OR (ambula* care)) AND (belgi*)

Databases: PubMed, Cochrane Reviews, TRIP Database, Epistemonikos, Web of Science

Date searched: 10/10/2016

Dates searched: All

Conclusion: use [Lorant et al. (2016)](#_ENREF_8), secondary analysis performed on patients diagnosed with schizophrenia

PRISMA diagram for the search is displayed in Figure A.5.

Figure A.5: PRISMA diagram of the Pragmatic Literature Review on the degree to which patients are hospitalized or in ambulatory care when in relapse.

## Probability of discontinuation for long-acting injections

Search terms [title/abstract]: schizophren* AND (discontin* OR adheren*) AND (inject*)

Databases: PubMed, Cochrane Reviews, TRIP Database, Epistemonikos, Web of Science

Date searched: 26/10/2016

Dates searched:

Web of Science, PubMed: 2013-2016

Cochrane reviews, TRIP Database, Epistemonikos: all dates

Conclusion: literature inconclusive, use primary data from [Decuypere et al. (2017)](#_ENREF_5).

PRISMA diagram for the search is displayed in Figure A.6.

Figure A.6: PRISMA diagram of the Pragmatic Literature Review on the relationship between the discontinuation of a long-acting injectable antipsychotic and the time between injections.

## Length of hospitalization in Belgium

Search terms [title/abstract]: (hospitali*) AND (schizophren*) AND (relaps*) AND (belgi*)

Databases: PubMed, Cochrane Reviews, TRIP Database, Epistemonikos, Web of Science

Date searched: 02/06/2016

Dates searched: All

Conclusion: primary data is more conservative than literature

PRISMA diagram for the search is displayed in Figure A.7.

Figure A.7: PRISMA diagram of the Pragmatic Literature Review on the length of hospitalization in Belgium.

## Length of relapse in hospital and ambulatory care

Search terms [title/abstract]: (schizophren*) AND (hospitali*) AND ((commun* care) OR (ambula* care)) AND (relaps*) AND (belgi*)

Databases: PubMed, Cochrane Reviews, TRIP Database, Epistemonikos, Web of Science

Date searched: 11/10/2016

Dates searched: All

Conclusion: literature is uninformative, assume same length

PRISMA diagram for the search is displayed in Figure A.8.

Figure A.8: PRISMA diagram of the Pragmatic Literature Review on the difference in length of relapse between hospitalization and ambulatory care in Belgium.

## Length of relapse and share of hospitalization

Search terms: schizophren*:ti AND (relaps*:ti,ab OR episod*:ti,ab OR recurren*:ti,ab OR deteriorat*:ti,ab OR recidiv*:ti,ab) AND (duration:ti,ab OR length:ti,ab) AND (hospital*:ti,ab)

Databases: Embase

Date searched: 10/11/2016

Dates searched: 2006-2016

Conclusion: literature inconclusive, use primary data.

PRISMA diagram for the search is displayed in Figure A.9.

Figure A.9: PRISMA diagram of the Pragmatic Literature Review on the difference in length of relapse between hospitalization and ambulatory care in Belgium.

## Probability of death

Search terms [title/abstract]: (schizophren*) AND (standardized mortality rat*) AND (mortality)

Databases: PubMed, Cochrane Reviews, TRIP Database, Epistemonikos, Web of Science

Date searched: 01/09/2016

Dates searched: All

Conclusion: use [Saha et al. (2007)](#_ENREF_11) for stable and [Hoang et al. (2011)](#_ENREF_6) for relapsed patients

PRISMA diagram for the search is displayed in Figure A.10.

Figure A.10: PRISMA diagram of the Pragmatic Literature Review on schizophrenia-specific mortality in the world.

# Life Cycle Assessment by Human Health midpoint category

The environmental Human Health burden in DALYs per midpoint category can be found in Table A.4.

Table A.4: Environmental Human Health burden in Disability-Adjusted Life Years (DALYs) per midpoint category, for 1000 patients for 1 year.

| **PP1M** | API | Drug Production | Packaging | Distribution & Supply | End-of-Life disposal & drug fate | GP visits | Psychiatrist visits | Ambulatory care visits | General hospital days | Psychiatric hospital days |
| --- | --- | --- | --- | --- | --- | --- | --- | --- | --- | --- |
| Climate Change | 5.98E-03 | 1.91E-03 | 2.72E-03 | 3.22E-05 | -3.05E-05 | 3.26E-03 | 2.24E-01 | 1.63E-03 | 2.42E-02 | 1.61E-01 |
| Human Toxicity | 4.36E-04 | 5.32E-05 | 2.99E-04 | 4.73E-06 | 7.31E-06 | 5.35E-04 | 3.67E-02 | 2.68E-04 | 2.17E-03 | 2.01E-02 |
| Ionizing Radiation | 1.58E-06 | 6.98E-07 | 2.90E-06 | -7.72E-08 | -6.29E-06 | 3.16E-06 | 2.17E-04 | 1.58E-06 | 2.65E-04 | 2.35E-03 |
| Ozone Depletion | 1.29E-06 | 4.83E-07 | 4.51E-07 | 9.37E-09 | -5.42E-08 | 9.55E-07 | 6.55E-05 | 4.78E-07 | 6.63E-06 | 7.42E-05 |
| Particulate Matter Formation | 1.83E-03 | 2.71E-04 | 7.11E-04 | 1.32E-05 | 1.72E-06 | 8.03E-04 | 5.50E-02 | 4.01E-04 | 3.91E-03 | 2.84E-02 |
| Photochemical Oxidant Formation | 3.96E-07 | 1.22E-07 | 2.46E-07 | 5.75E-09 | 4.73E-09 | 2.55E-07 | 1.75E-05 | 1.28E-07 | 1.18E-06 | 9.47E-06 |
|  |  |  |  |  |  |  |  |  |  |  |
| **PP3M** |  |  |  |  |  |  |  |  |  |  |
| Climate Change | 7.60E-03 | 2.42E-03 | 1.09E-03 | 1.40E-05 | -1.13E-05 | 3.38E-03 | 2.25E-01 | 8.63E-04 | 1.29E-02 | 8.54E-02 |
| Human Toxicity | 5.54E-04 | 6.73E-05 | 1.26E-04 | 2.06E-06 | 1.60E-05 | 5.55E-04 | 3.70E-02 | 1.42E-04 | 1.15E-03 | 1.07E-02 |
| Ionizing Radiation | 2.01E-06 | 8.86E-07 | 1.36E-06 | -3.36E-08 | -2.34E-06 | 3.28E-06 | 2.19E-04 | 8.37E-07 | 1.41E-04 | 1.25E-03 |
| Ozone Depletion | 1.64E-06 | 6.13E-07 | 1.81E-07 | 4.09E-09 | -2.01E-08 | 9.91E-07 | 6.61E-05 | 2.53E-07 | 3.52E-06 | 3.93E-05 |
| Particulate Matter Formation | 2.33E-03 | 3.44E-04 | 2.88E-04 | 5.76E-06 | 6.41E-07 | 8.33E-04 | 5.55E-02 | 2.13E-04 | 2.08E-03 | 1.50E-02 |
| Photochemical Oxidant Formation | 5.03E-07 | 1.55E-07 | 9.70E-08 | 2.51E-09 | 1.76E-09 | 2.65E-07 | 1.77E-05 | 6.77E-08 | 6.24E-07 | 5.02E-06 |
|  |  |  |  |  |  |  |  |  |  |  |
| **TI** |  |  |  |  |  |  |  |  |  |  |
| Climate Change | N/A | N/A | N/A | N/A | N/A | 2.83E-03 | 2.16E-01 | 4.30E-03 | 5.72E-02 | 4.31E-01 |
| Human Toxicity | N/A | N/A | N/A | N/A | N/A | 4.64E-04 | 3.55E-02 | 7.06E-04 | 5.13E-03 | 5.37E-02 |
| Ionizing Radiation | N/A | N/A | N/A | N/A | N/A | 2.74E-06 | 2.10E-04 | 4.17E-06 | 6.26E-04 | 6.28E-03 |
| Ozone Depletion | N/A | N/A | N/A | N/A | N/A | 8.29E-07 | 6.34E-05 | 1.26E-06 | 1.57E-05 | 1.98E-04 |
| Particulate Matter Formation | N/A | N/A | N/A | N/A | N/A | 6.96E-04 | 5.33E-02 | 1.06E-03 | 9.23E-03 | 7.58E-02 |
| Photochemical Oxidant Formation | N/A | N/A | N/A | N/A | N/A | 2.22E-07 | 1.70E-05 | 3.37E-07 | 2.77E-06 | 2.53E-05 |

Abbreviations: PP1M, paliperidone palmitate once-monthly injection; PP3M, paliperidone palmitate three-monthly injection; TI, Treatment Interruption; GP, General Practitioner; N/A, Not Applicable

# One-way sensitivity analysis

The sensitivity of the base case results was explored through a One-way Sensitivity Analysis, which was performed using Excel macro’s. The One-way Sensitivity Analysis presents the Human Health benefit in QALYs gained or DALYs avoided of the PP1M and PP3M care pathways versus TI.

The key parameters were varied by the standard deviation or ±20% if that information was not available. The Belgian cost-effectiveness guidelines do not propose a specific percentage for parameter variation ([Cleemput et al. 2012](#_ENREF_4)). The value of ±20% was considered because the standard deviation of the other parameters approximated this value.

The parameters are the PP1M and PP3M monthly relapse probability, PP1M and PP3M monthly relapse probability after discontinuation, PP1M and PP3M discontinuation probability, TI monthly relapse probability, percentage of patients requiring hospitalization during relapse, probability of going to the Adherent state from the Non-adherent state and the probability of going to Stable: Adherent from a relapse state.

For the length of relapse this was less straightforward as tunnel states are used. The probability of being discharged from one of the hospitalized states was varied by 50%. This caused the length of relapse to vary for +13.16% up and -12.62% down.

The results are displayed in Figure A.11 and Figure A.12. The length of relapse is the most sensitive parameter.

In a separate sensitivity analysis we ran the model for a 2-year time horizon. The results show that a 1-year time horizon is more conservative.


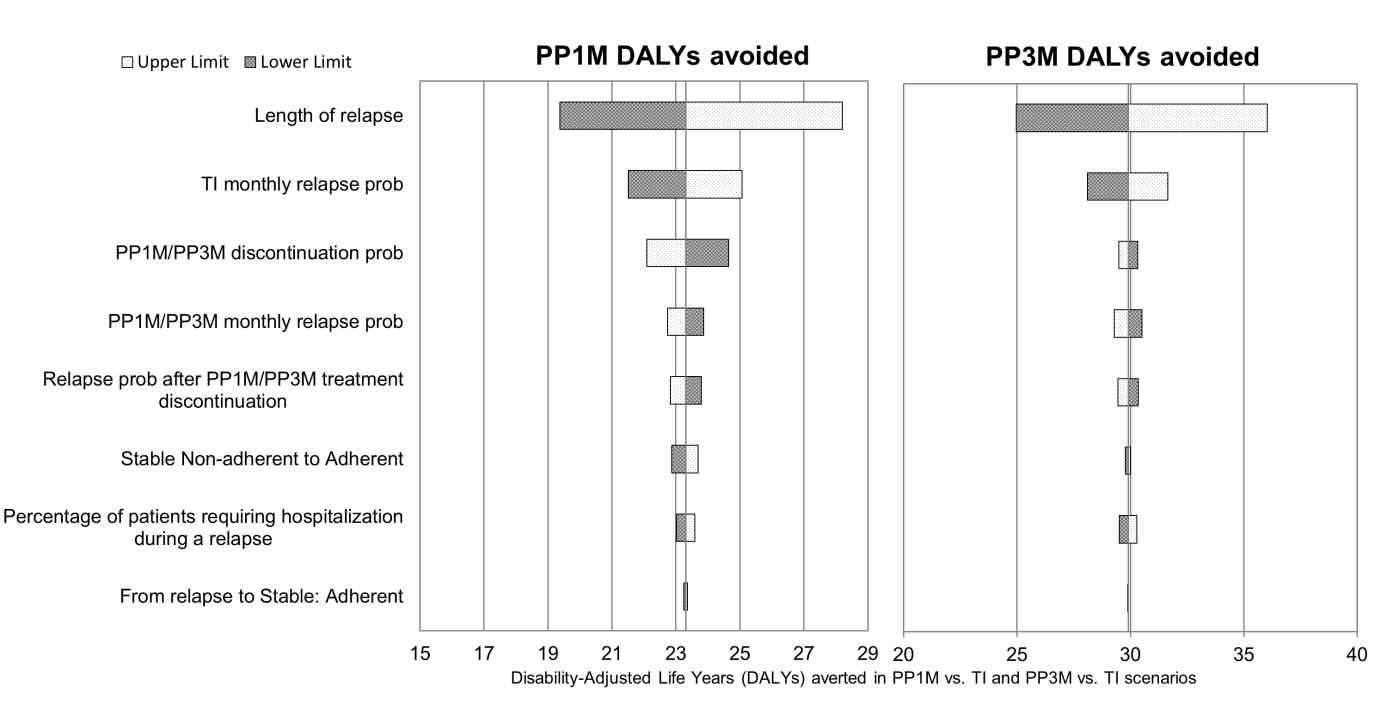


Figure A.11: Tornado diagram displaying the sensitivity of the model to key input parameters. The outcomes presented by the bar graphs are the Disability-Adjusted Life Years (DALYs) avoided by the paliperidone palmitate 1M (PP1M) and paliperidone palmitate 3M (PP3M) care pathways versus Treatment Interruption (TI).


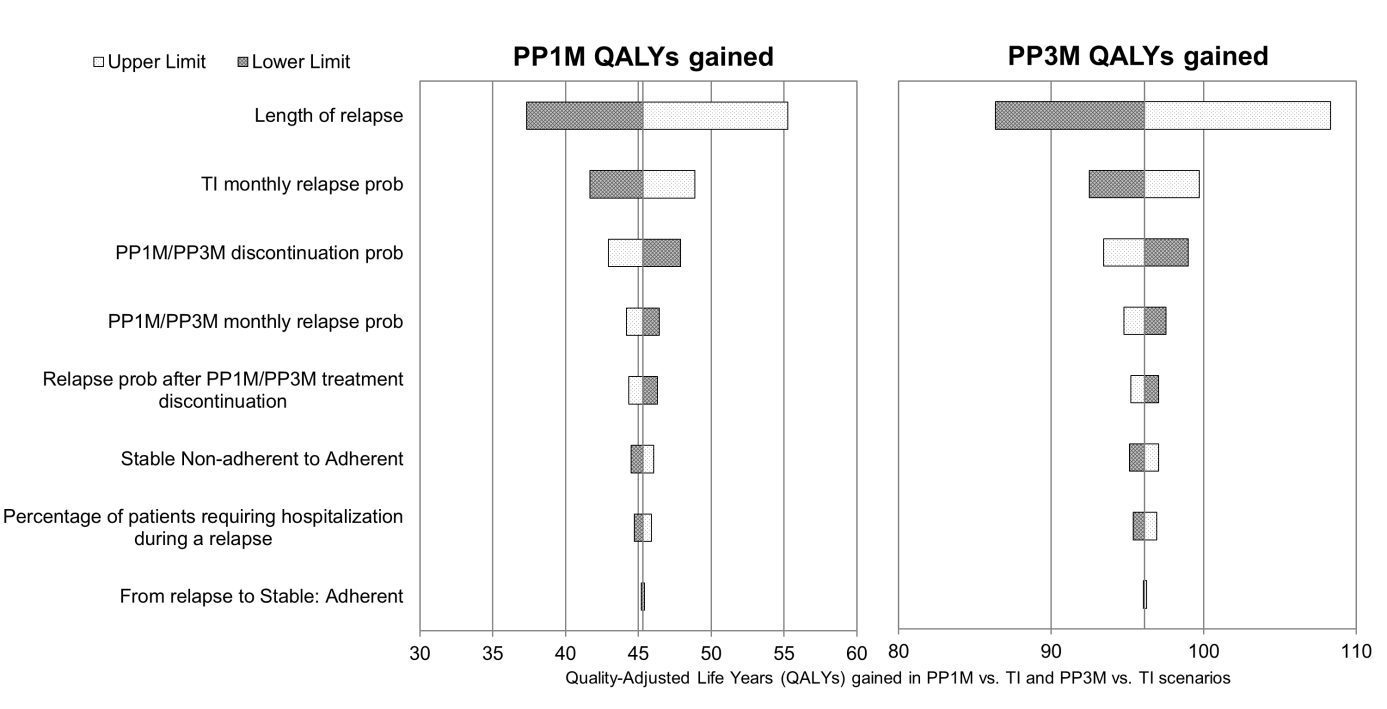


Figure A.12: Tornado diagram displaying the sensitivity of the model to key input parameters. The outcomes presented by the bar graphs are the Quality-Adjusted Life Years (QALYs) gained by the paliperidone palmitate 1M (PP1M) and paliperidone palmitate 3M (PP3M) care pathways versus Treatment Interruption (TI).

# Probabilistic sensitivity analysis

The Probabilistic Sensitivity Analysis (PSA) includes the same parameters as the One-way Sensitivity Analysis and was also performed using Excel macro’s. The PSA varied the probabilities for relapse and discontinuation though beta and normal distributions. The percentage of patients requiring hospitalization, the Stable Non-adherent to Adherent and from relapse to Stable: Adherent were randomly varied with a maximum relative margin of 20% ([Briggs et al. 2006](#_ENREF_2)). For the tunnel states that define the length of relapse we varied the probability of being discharged from one of the hospitalized states by 50%.

We calculated the environmental Human Health burden for each iteration, because the patient consumption profile is different each time.

The PSA ran for 10,000 iterations and the results are displayed in Figure A.13. The patient health benefits are expressed in QALYs with the aim to avoid confusion in the figure by using DALYs on both the x and y axis.


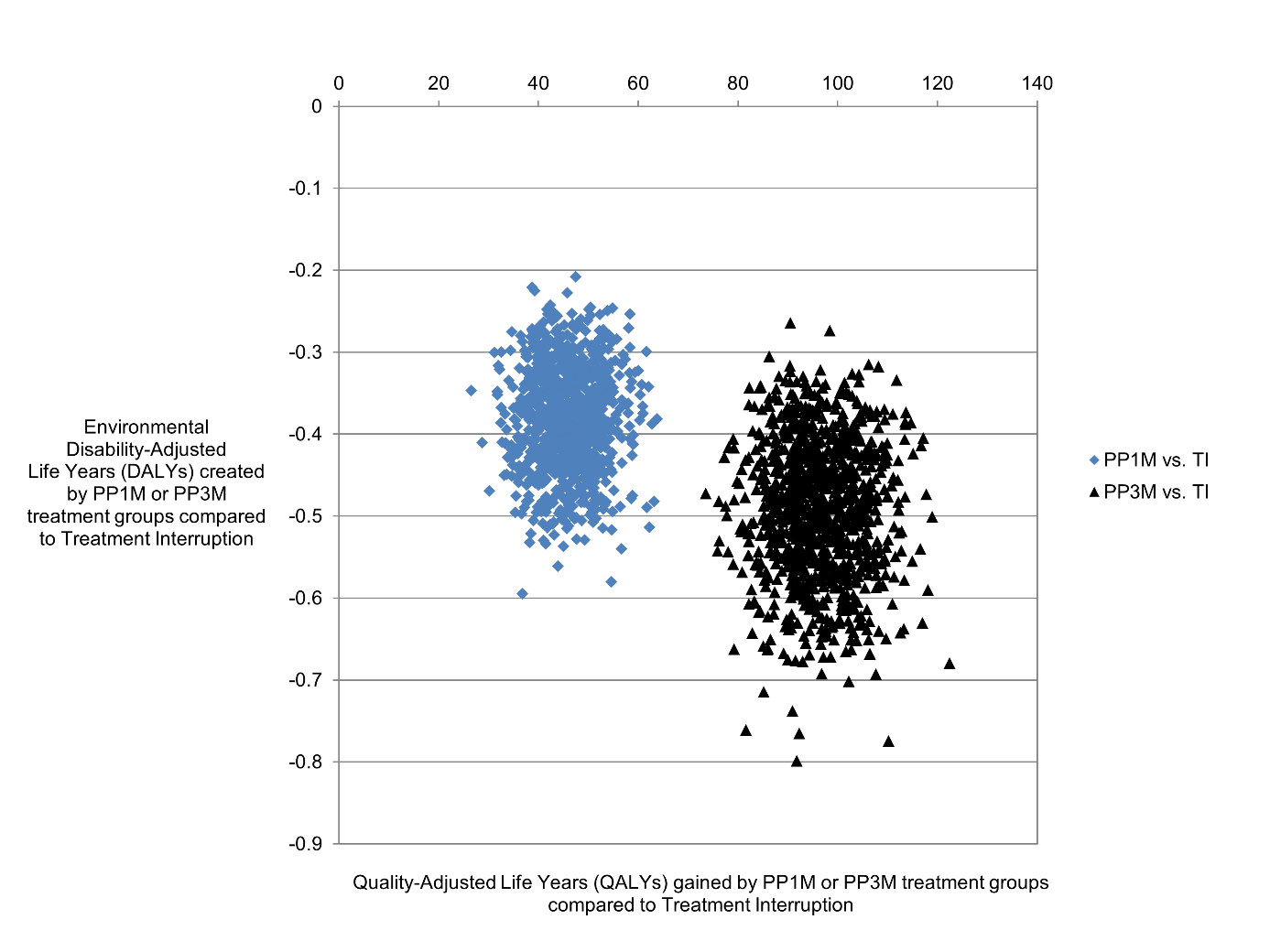


Figure A.13: Environmental burden vs. disease outcomes of Paliperidone Palmitate 1M (PP1M) and Paliperidone Palmitate 3M (PP3M) care pathways vs. Treatment Interruption (TI). The gains in QALYs for the patient are compared to the avoided (negative) environmental DALYs, representing a benefit in both cases. The Probabilistic Sensitivity Analysis (PSA) ran for 10,000 iterations.

# References

Berwaerts J, Liu Y, Gopal S, et al. 2015. Efficacy and safety of the 3-month formulation of paliperidone palmitate vs placebo for relapse prevention of schizophrenia: A randomized clinical trial. JAMA Psychiatry 72:830-839.

Briggs A, Claxton K, Sculpher M. 2006. Decision Modelling for Health Economic Evaluation. US: Oxford University Press.

Briggs A, Wild D, Lees M, Reaney M, Dursun S, Parry D, et al. 2008. Impact of schizophrenia and schizophrenia treatment-related adverse events on quality of life: direct utility elicitation. Health Qual Life Outcomes 6:105.

Cleemput I, Neyt M, Van De Sande S, Thiry N. 2012. Belgian guidelines for economic evaluations and budget impact analysis: second edition Belgian Health Care Knowledge Centre (KCE).

Decuypere F, Sermon J, Geerts P, Denee TR, De Vos C, Malfait B, et al. 2017. Treatment continuation of four long-acting antipsychotic medications in the Netherlands and Belgium: A retrospective database study. PLoS One 12:e0179049.

Hoang U, Stewart R, Goldacre MJ. 2011. Mortality after hospital discharge for people with schizophrenia or bipolar disorder: retrospective study of linked English hospital episode statistics, 1999-2006. BMJ 343:1-13.

Hough D, Gopal S, Vijapurkar U, Lim P, Morozova M, Eerdekens M. 2010. Paliperidone palmitate maintenance treatment in delaying the time-to-relapse in patients with schizophrenia: A randomized, double-blind, placebo-controlled study. Schizophr Res 116:107-117.

Lorant V, Grard A, Van Audenhove C, Helmer E, Vanderhaegen J, Nicaise P. 2016. Assessment of the priority target group of mental health service networks within a nation-wide reform of adult psychiatry in Belgium. BMC Health Serv Res 16:187.

Marcus SC, Zummo J, Pettit AR, Stoddard J, Doshi JA. 2015. Antipsychotic Adherence and Rehospitalization in Schizophrenia Patients Receiving Oral Versus Long-Acting Injectable Antipsychotics Following Hospital Discharge. J Manag Care Spec Pharm 21:754-768.

Osborne RH, Dalton A, Hertel J, Schrover R, Smith DK. 2012. Health-related quality of life advantage of long-acting injectable antipsychotic treatment for schizophrenia: a time trade-off study. Health Qual Life Outcomes 10:1-9.

Saha S, Chant D, McGrath J. 2007. A systematic review of mortality in schizophrenia: Is the differential mortality gap worsening over time? Arch Gen Psychiat 64:1123-1131.

Salomon JA, Haagsma JA, Davis A, de Noordhout CM, Polinder S, Havelaar AH, et al. 2015. Disability weights for the Global Burden of Disease 2013 study. Lancet Glob Health 3:e712-e723.

Savitz AJ, Xu H, Gopal S, Nuamah I, Ravenstijn P, Janik A, et al. 2016. Efficacy and Safety of Paliperidone Palmitate 3-Month Formulation for Patients with Schizophrenia: A Randomized, Multicenter, Double-Blind, Noninferiority Study. Int J Neuropsychopharmacol 19:1-14.

Statbel. 2015. Sterftetafels en levensverwachting. Available: <http://statbel.fgov.be/nl/statistieken/cijfers/bevolking/sterfte_leven/tafels/> [accessed 17 February 2016].
